# Comparative analysis of different modalities of radiotherapy in vestibular schwannoma: tumor control, symptom evolution, and toxicity profiles

**DOI:** 10.1007/s00066-026-02516-1

**Published:** 2026-03-27

**Authors:** Phillipp Lishewski, Maike Fischer, Kerem Tuna Tas, Fatima Frosan Sheikhzadeh, Edgar Smalec, Linda Agolli, Christopher Nimsky, André Kemmling, Daniel Habermehl, Klemens Zink, Ahmed Gawish, Sebastian Adeberg

**Affiliations:** 1 Department of Radiotherapy and Radiation Oncology, Philipps-Universität Marburg, Marburg, Germany; 2 Department of Radiotherapy and Radiation Oncology, Marburg University Hospital, Marburg, Germany; 3 Marburg Ion-Beam Therapy Center (MIT), Department of Radiotherapy and Radiation Oncology, Marburg University Hospital, Marburg, Germany; 4 University Cancer Center (UCT) Frankfurt—Marburg, Marburg, Germany; 5 LOEWE Research Cluster for Advanced Medical Physics in Imaging and Therapy, (ADMIT), TH Mittel Hessen University of Applied Sciences, Giessen, Germany; 6 Department for Neurosurgery, Marburg University Hospital, Marburg, Germany; 7 Department for Neuroradiology, Marburg University Hospital, Marburg, Germany; 8 Department of Radiotherapy and Radiation Oncology, Giessen University Hospital, Giessen, Germany

**Keywords:** Proton therapy, Radiosurgery, Fractionated radiotherapy, Hearing preservation, Cranial nerve outcomes

## Abstract

**Background:**

Stereotactic radiotherapy (SRT) is a standard treatment for vestibular schwannoma (VS). Long-term comparative data on single-fraction radiosurgery (SRS) versus normofractionated SRT (NFSRT) are limited.

**Methods:**

We retrospectively analyzed 175 VS patients treated between 1998 and 2023 (SRS *n* = 69; NFSRT *n* = 106; median age 61 years; median follow-up 46 months). Tumor control was evaluated with Kaplan–Meier and Cox regression. Group differences were tested with Mann–Whitney U and chi-square/Fisher’s exact tests. Symptom changes were assessed with McNemar tests, and binary logistic regression identified predictors of acute and late toxicity.

**Results:**

Overall, in-field PFS was 94.3% and out-field was PFS was 99.4%. All 10 recurrences (median 58.5 months) occurred in NFSRT patients treated with 55.8–56 Gy. Higher total dose predicted recurrence (HR = 2.97; *p* = 0.003). At baseline, 97.1% reported symptoms (hearing loss 86.9%, vertigo 48.0%, tinnitus 42.3%). After therapy, symptoms remained stable, except the incidence of headache, which increased from 14.3% to 22.3% (*p* = 0.02). Early toxicities were more common after NFSRT, including headache (OR = 4.05; *p* = 0.01), fatigue (OR = 5.12; *p* = 0.01), and alopecia (OR = 19.9; *p* = 0.04), but had resolved by late follow-up. Age and prior surgery predicted vertigo patterns over time. Radionecrosis was rare (0.6%).

**Conclusion:**

Both SRS and NFSRT achieve excellent long-term tumor control with low severe toxicity. For NFSRT, normofractionated doses above 55 Gy did not improve efficacy and may increase the recurrence risk, supporting moderate-dose regimens (50–54 Gy). Symptom stabilization confirms the safety of SRT, though headache and vertigo remain relevant long-term issues. Careful dose selection, patient counseling, and rehabilitation are essential to optimize outcomes.

## Introduction

Vestibular schwannoma (VS) is a predominantly benign, slow-growing tumor arising from the Schwann cells of the vestibular portion of the eighth cranial nerve (CN VIII) within the internal auditory canal or cerebellopontine angle [1]. Historically classified as a rare neoplasm with an incidence of approximately 1.2 per 100,000 person-years, advancements in diagnostic imaging have revised this understanding [2]. Contemporary epidemiological studies suggest that the true lifetime prevalence is significantly higher, now estimated to be nearly 1 in 500 individuals [3, 4]. This increased detection is largely attributable to greater physician awareness and the widespread availability of high-resolution magnetic resonance imaging (MRI), facilitating diagnosis at earlier, often asymptomatic or paucisymptomatic stages [3–5].

The clinical presentation of VS is characterized by symptoms stemming from its anatomical location. The most common initial manifestation is unilateral sensorineural hearing loss, frequently accompanied by tinnitus and vertigo or disequilibrium, which may be present for years prior to formal diagnosis [6]. As the tumor progresses, it can exert a mass effect on adjacent neural structures, leading to more severe complications such as facial nerve palsy (CN VII), trigeminal neuralgia (CN V), and, in neglected cases, brainstem compression and hydrocephalus [6]. Modern management of VS has evolved into a multidisciplinary paradigm. Treatment options include surgical resection; stereotactic radiotherapy (SRT); and active surveillance with serial imaging for selected, asymptomatic patients with small tumors and stable symptoms [7].

For patients requiring active intervention, stereotactic radiotherapy is a cornerstone treatment, particularly for tumors of modest size (typically < 3 cm in the largest diameter) or in cases where advanced age, comorbidities, or patient preference render surgical risks prohibitive [7, 8]. Radiotherapy can be delivered as single-fraction stereotactic radiosurgery (SRS) or as fractionated stereotactic radiotherapy (FSRT), which includes hypofractionated (HFRT) and normofractionated (NFRT) regimens [9]. Normofractionated RT typically involves total doses ranging from 45 to 57.6 Gy delivered in 20 to 32 fractions [9, 10]. While long-term tumor control rates exceeding 90% at 10 years are consistently achieved with both SRS and FSRT, the choice of modality is often guided by a desire to preserve neurological function, particularly useful hearing and facial nerve function [11, 12]. A foundational radiobiological principle suggests that fractionated schedules exploit the differential repair capacities of neoplastic and normal neural tissues, potentially offering a superior therapeutic ratio for functional preservation [12, 13]. Consequently, FSRT is frequently preferred for larger tumors (> 2–3 cm) or for any patient with serviceable hearing at diagnosis [7, 11].

However, the comprehensive comparison between these modalities regarding long-term efficacy, detailed symptom evolution, and nuanced toxicity profiles, particularly of NFRT, remains an area of active investigation. Robust data identifying clear predictors of treatment outcomes are still needed to optimize personalized therapy. This study aims to perform a comparative analysis of SRS and NFRT in a large single-institution cohort of VS patients. Our primary objectives are to evaluate predictors of tumor control and safety, including the incidence of adverse events (AEs), and to meticulously track the long-term trajectory of VS-related symptoms before and after radiotherapy. Ultimately, this work seeks to identify specific patient and treatment characteristics predictive of optimal oncological and functional outcomes, thereby contributing to more refined and evidence-based management strategies for vestibular schwannoma.

## Materials and methods

### Patient collective

After approval from our ethics committee (nr. 24-138-RS on 03.06.2024), we retrospectively identified 190 patients with vestibular schwannoma between December 1998 and October 2023 at the Department of Radiooncology and Radiotherapy at the University Hospital Marburg. We only included patients with a minimum follow-up (FU) of 6 months and without previous radiotherapy to the site of the current VS or any malignancies at the time of SRT. We excluded patients with insufficient FU or with active malignancies as well as patients who had schwannoma at any other site than CN VIII. Also, patients with HFSRT, defined as RT in 2–10 fractions, were excluded from the analysis, since this fractionation schedule was not routinely used at our department before 2022. Lastly, all patients with sufficient follow-up examinations (MRI scans and presentation in our facility for clinical evaluation of effects and side effects) were included. Thus, the final cohort consisted of 175 patients, all with complete information regarding toxicities and recurrences. Only the PTV was not retrievable in all cases (97/175 had sufficient information) due to a change of our planning system in 2014. A neurooncological tumor board had evaluated all patients before therapy. Patients with large VS, especially affecting the brain stem (BS), or with accessible VS wishing for primary surgery were voted in favor of surgery. In this case, patients with subtotal resection or tumor regrowth were referred to SRT afterwards. In all other cases, primary SRT was performed.

### Irradiation protocol

Stereotactic radiotherapy planning utilized computed tomography (CT) supplemented by contrast-enhanced magnetic resonance imaging (MRI), preferably aided by the inclusion of constructive interference in steady state (CISS) or Fast Imaging Employing Steady-State Acquisition (FIESTA) sequences. Thermoplastic masks were individually fitted and employed across the CT and radiotherapy tables to ensure precise isocenter localization. Linear accelerators were used to deliver the SRT. The isocenter position was verified before each treatment session. Stereotactic radiosurgery was preferred for smaller VS or for larger VS with higher symptomatic burden at the time of treatment. All other cases were selected for HFSRT. However, institutional standards were subjected to changes over the course of the FU period, and deviations from this approach were possible. For SRS, GTV to PTV margins of 1–2 mm were used. For HFSRT, margins of 3–4 mm were used to generate the PTV. The dose was prescribed to the 80% isodose.

### Follow-up regimen

The first clinical FU occurred after 6 weeks. Three months after SRT, the first MRI scan was performed. Thereafter, two scans took place within 6 months. After that, re-staging took place every 12–24 months, depending on the patient’s age, symptoms, and previous findings. In cases of irregular MRI findings, earlier control MRIs were encouraged; MRI follow-up and response assessment were performed according to the Response Evaluation Criteria in Solid Tumors (RECIST) [14], although in patients case who progressed in 2005, that had to be done retrospectively. All imaging studies were reviewed in our department and discussed in a multidisciplinary neurooncological tumor board. To reduce false-positive findings (e.g., transient swelling or cystic changes), progression was confirmed only if radiological progression was also evident on subsequent follow-up imaging, even if the RECIST criteria were fulfilled [15–17]. Radionecrosis was also assessed interdisciplinarily and was assumed if contrast-enhancing lesions, possibly accompanied by an edema, were found that conformed to the prior high-dose radiotherapy area [17, 18].

### Statistical analysis

For statistical analysis, SPSS version 29 from IBM (Armonk, NY, USA) [19] was used with a significance level set to *p* < 0.05. For metric variables, the Shapiro–Wilk test was used to check for a normal distribution. Thus, the Mann–Whitney U test was performed to check for imbalances between patients undergoing primary vs. adjuvant SRT. For categorical variables, this was tested using chi-square tests or Fisher’s exact tests in cases with < 5 cell counts in each cross table. The Kaplan–Meier method was employed to depict in-field progression-free survival (IFPFS) and the graphic was built in GraphPad Prism (GraphPad Software, Inc. (Dotmatics), San Diego, California) [20]. In-field progression (IFPFS) was defined as progression within the PTV. Out-field progression-free survival (OFPFS) was defined as any progression or secondary cancer forming outside the PTV following RT. Individual and Cox testing was performed to identify predictors of these clinical endpoints. Multivariate Cox proportional hazard models were omitted due to the low number of events. Pre- and post-therapeutic symptoms were recorded dichotomously before SRT (T0), at the first FU 6 weeks after SRT (T1), and until the end of FU (T2). Binary logistic regression was used to screen for predictors of post-therapeutic VS-related symptoms at T1 and T2, and the McNemar test was employed to identify significant changes in pre- (T0) and post-therapeutic symptoms (T2).

## Results

**Fig. 1:**
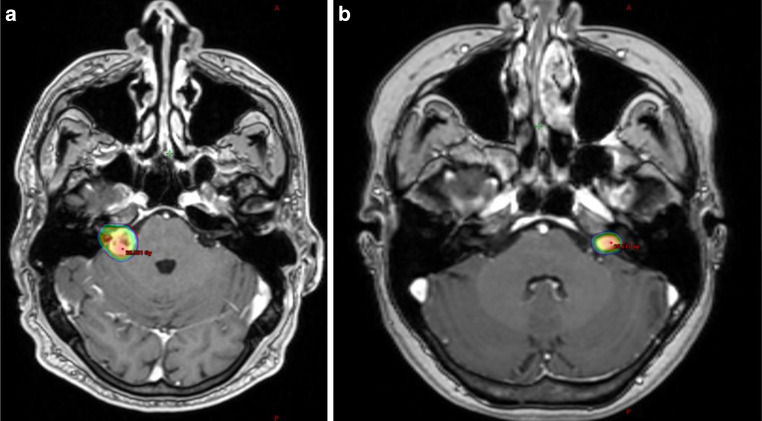
Exemplary dose distributions for patients with NFSRT (normofractioned Radiotherapy) (left) and SRS (Radiosurgery) (right)

A total of 175 patients were included in this study. Median age at the time of SRT was 61 years (range 14–84 years; SD ± 14.2). We included 91 male (52%) and 84 female (48%) patients. Lesions were evenly distributed between the right (*n* = 91, 52%) and left (*n* = 84, 48%) CN VIII. While 69 patients (39.4%) underwent SRS, 106 patients (60.6%) received NFSRT. In the latter group, the median number of fractions was 31 (range 14–34; SD ± 3.1), with a median total dose of 55.8 Gy (range 42.0–59.4 Gy; SD ± 2.3 Gy). Representative dose distributions for NFSRT and SRS treatment plans are shown in Fig. 1, demonstrating the higher conformity and steeper dose gradients achieved with stereotactic techniques.

In the SRS group, the median dose was 13 Gy (range 12–15 Gy; SD ± 0.84 Gy). While 138 patients (78.9%) received primary SRT, partial resection (PR) had been performed in 37 patients (21.1%), of whom 32 had undergone a single surgical procedure (86.5%) and 5 had undergone two resections (13.5%). Of 37 resected patients, 24 received NFSRT, the rest underwent SRS. The median planning target volume (PTV) was 1.26 cm^3^ (range 0.05–32.6 cm^3^; SD ± 5.6 cm^3^). Median follow-up duration was 46 months (range 6–282 months; SD ± 54.9). Table 1 provides a detailed overview of our collective. Out of 175 patients, 170 (97.1%) reported symptoms prior to SRS. Most patients reported impairments of useful hearing prior to SRT (*n* = 152, 86.9%). After that, the most prevalent symptoms were vertigo in 84 patients (48.0%) and tinnitus in 74 (42.3%). More symptoms and their distribution throughout T0–T2 are found in Table 2. Note, however, that at T1, only few symptoms were retrievable in sufficient quality. At T2, hearing loss, vertigo, and tinnitus remained the most common symptoms.

**Table 1:** Characteristics of our cohort of 175 patients

Age, median (SD; range)	61 (± 14.2; 14–84)
**Sex, number (%)**	
*Male*	91 (52%)
*Female*	84 (48%)
**Site, number (%)**	
*Right*	91 (52%)
*Left*	84 (48%)
**SRT form, number (%)**	
*SRS*	69 (39.4%)
*NFSRT*	106 (60.6%)
Fx number for NFSRT, median (range; SD)	31 (3.1; 14–34)
NFSRT dose, median (range; SD)	55.8 Gy (42.0 Gy–59.4 Gy; ±2.3)
SRS dose, median (range; SD)	13 Gy (12 Gy–15 Gy; ±0.84 Gy)
**Symptoms before SRS, number (%)**	
*No*	5 (2.9%)
*Yes*	170 (97.1%)
**Treatment before SRS, number (%)**	
*None*	138 (78.9%)
*Partial resection*	37 (21.1%)
*One resection*	32 (86.5%)
*Two resections*	5 (13.5%)
PTV, median, known in *n* = 97 cases (SD; range)	1.26 cm^3^ (5.6 cm^3^; 0.05 cm^3^–32.6 cm^3^)
Follow-up, median (SD; range)	46 (54.9; 6–282)

*SD* standard deviation, *SRT* stereotactic radiotherapy, *SRS* stereotactic radiosurgery, *NFSRT* normofractionated stereotactic radiotherapy, *PTV* planning target volume, *Fx* fraction

**Table 2:** Symptomatology of the cohort of *n* = 175 patients before SRT (baseline, T0), at first FU (T1), and until the end of FU (T2)

	Baseline, T0	Acute, T1	Late, T2
	Number (%)	Number (%)	Number (%)
Asymptomatic	5 (2.9%)	100 (57.1%)	14 (8.0%)
Headache	25 (14.3%)	28 (16.0)	39 (22.3%)
Hear loss	152 (86.9%)	n/a	154 (88.0%)
Tinnitus	74 (42.3%)	n/a	61 (34.9%)
Vertigo	84 (48.0%)	10 (5.7%)	75 (42.8%)
Ataxia	13 (7.4%)	n/a	9 (5.1%)
Nausea	6 (3.4%)	8 (4.6%)	3 (1.7%)
Vomiting	3 (1.7%)	0	1 (0.6%)
Trigeminal neuralgia	2 (1.1%)	n/a	5 (2.9%)
Facial paralysis	13 (7.4%)	n/a	12 (6.9%)
Fatigue	1 (0.6%)	23 (13.1%)	0
Alopecia	n/a	25 (14.3%)	0
Radionecrosis	n/a	n/a	1 (0.6%)

Asymptomatic not assessed at T1 due to missing data in parts of the variables

*FU* follow-up

**Fig. 2:**
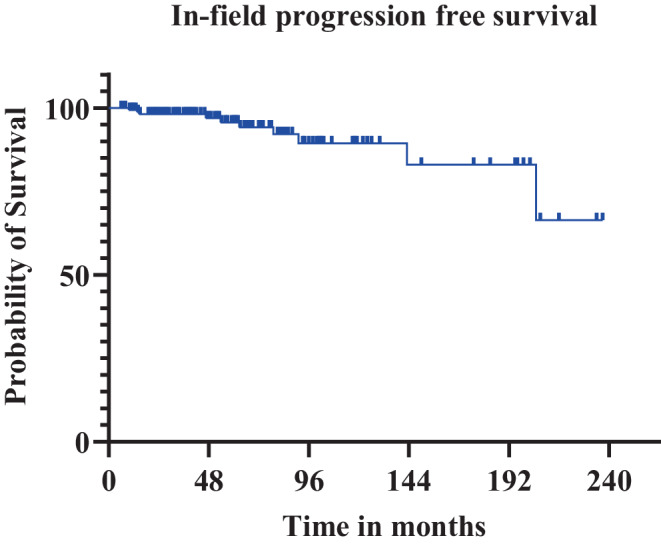
Kaplan–Maier estimation of the IFPFS rate after SRT for VS

Over the course of our FU period, 10 cases of IFP were observed after 10, 14, 15, 47, 54, 63, 79, 91, 143, and 205 months, respectively (median 58.5 months), leading to an IFPFS rate of 94.3%. Meanwhile, one patient developed a meningioma 39 months after SRT without IFP. Thus, the OFPFS rate stood at 99.4%. Figure 2 depicts the Kaplan–Meyer estimation for IFPFS. All progressive cases were confirmed either histologically following salvage surgery or were evaluated as progressive following progression in consecutive MRI scans to avoid false-positive findings due to pseudoprogression or cystic degeneration. The influence of the following factors on IFP rates was tested using univariate Cox proportional hazard models: age; gender; tumor site; prior PR; PTV; RT form (SRS vs. NFSRT); SRS dose over 13.0 Gy in the case of SRS; and dose per fraction, total dose, and number of fractions for cases where NFSRT was employed. Notably, we found that for NFSRT, increasing doses predicted higher IFP rates (HR = 2.97, 95% CI: 1.39–5.2; *p* = 0.003). On further inspection, we found that all cases of IFP occurred in patients treated with doses between 55.8 and 56 Gy in 1.8 Gy–2.0 Gy dose/fraction. However, out of the 106 patients treated with NFSRT, 81 received a dose of 55.8 Gy. Other than that, no significant predictors of IFP were found. Multivariate analyses of IFP and univariate analyses of OFP were not performed due to low event rates. Prior to this analysis, Mann–Whitney U tests had revealed no significant difference in the age distribution between patients treated with SRS vs. NFSRT (U = 3556.5; *p* = 0.76) and showed that patients treated with NFSRT had significantly larger PTVs (U = 1717; *p* < 0.001; Shapiro–Wilk tests had shown that both variables had a nonnormal distribution). Lastly, a Spearman test revealed no significant associations between tumor volumes and the prescribed NFSRT doses (ρ = 0.13; *p* = 0.39). Likewise, a chi-square test showed no significant inhomogeneity (χ^2^(1) = 0.36; *p* = 0.55) in terms of prior surgery between the groups.

The second part of our analysis focused on the symptomology of VS patients before and after SRT. McNemar’s tests only revealed a significant increase in the headache prevalence from baseline (T0) to the end of follow-up (T2) (χ^2^(1) = 5.28; *p* = 0.02). For all other symptoms, no significant changes were found between baseline symptoms and symptoms at the end of FU. Following this, binary logistic regression models were established to identify predictors of post-SRT symptoms at T1 and T2. At T1, increasing age correlated with a lower incidence of vertigo (OR = 0.95, 95% CI: 0.91–0.99; *p* = 0.018), and NFSRT predicted higher odds of headache (OR = 2.75, 95% CI: 1.05–7.18, *p* = 0.04). Likewise, application of NFSRT predicted higher odds of fatigue (OR = 5.12, 95% CI: 1.46–17.95; *p* = 0.01) and alopecia (OR = 19.9, 95% CI: 2.64–150.9; *p* = 0.04) at T1. Under multivariate control for PTV, the effects of age on the prevalence of vertigo (OR = 0.93, 95% CI: 0.88–0.98; *p* = 0.01) and of NFSRT on the occurrence of headache (OR = 4.05, 95% CI: 1.27–12.82; *p* = 0.01) remained significant, whereas the effects of NFSRT on fatigue and alopecia were no longer significant. At T2 and in univariate analysis, age correlated with a lower liklihood of tinnitus (OR = 0.96, 95% CI: 0.94–0.98; *p* = 0.01) but was predictive of vertigo (OR = 1.03, 95% CI: 1.0–1.05; *p* = 0.02). Other than that, patients who had received surgery were prone to vertigo at T2 (OR = 0.3, 95% CI: 0.13–0.69; *p* = 0.005). Again, correcting for PTV, the impact of age on tinnitus was rendered insignificant, while the influence of age on vertigo remained significant (OR = 1.03, 95% CI: 1.0–1.06; *p* = 0.04). Additionally, using chi-square tests, normofractionated SRT did not affect the incidence of the individual post-RT symptoms or the aggregated incidence of trigeminal or facial nerve impairments.

## Discussion

This retrospective analysis of 175 patients treated with stereotactic radiotherapy for vestibular schwannoma provides a robust long-term assessment of tumor control, symptomatic evolution, and toxicity profiles, with a particular focus on comparing single-fraction radiosurgery (SRS) and normofractionated stereotactic radiotherapy (NFSRT). With a median follow-up of 46 months, our data affirm the high efficacy of modern SRT while unveiling nuanced insights into dose–response relationships, symptom dynamics, and the late effects of treatment. These findings contribute significantly to the ongoing refinement of personalized therapeutic strategies for VS.

The cornerstone of any radiotherapy treatment is durable tumor control. Our study demonstrates exemplary outcomes in this regard, with an in-field progression-free survival (IFPFS) rate of 94.3% and an out-field progression-free survival (OFPFS) rate of 99.4% over the follow-up period. These figures are squarely in line with modern published series for both SRS and FSRT with photon and proton irradiation, which consistently report 5‑ and 10-year control rates exceeding 90%. Table 3 provides an overview of existing data from the past two decades using various fractionation schemes as well as photon and proton radiotherapy. Across numerous institutions and techniques, SRT provides control rates comparable to those of microsurgery but with a different profile of associated risks and benefits [7].

**Table 3:** Overview of published data for SRT for VS, including different treatment modalities and fractionation schemes

	*n*	*LC*	Modality	Follow-up
Lunsford et al. [21]	829	98.1%	GKS	72 months
Anurag et al. [22]	20	100%	NFPRT	48 months
Yildirim et al. [23]	104	89%	GKS	36 months
Combs et al. [24]	449	94%	SRS, NFSRT	60 months
Fuss et al. [20]	51	97.7%	HFSRT	60 months
Meijer [25]	129	100% (SRS, *n* = 49), 94% (FSRT, *n* = 80)	SRS, HFSRT	33 months
Kondziolka et al. [17]	285	93.7%	SRS	180 months
Sawamura et al. [26]	101	91.7%	NFSRT	60 months
Marchetti et al. [27]	100	92%	SRS/HFSRT	62 months
Gawish et al. [28]	134	94	HFRT	54 months

*n* number of patients included, *LC* local control, *GKS* Gamma Knife® radiosurgery, *NFPRT* normofractionated proton therapy, *NFSRT* normofractionated stereotactic radiotherapy, *HFSRT* hypofractionated stereotactic radiotherapy, *SRS* stereotactic radiosurgery

However, the most intriguing and potentially practice-informing finding of our analysis is the apparent dose–response relationship observed within the NFSRT cohort. Contrary to the conventional oncological principle that higher radiation doses yield superior tumor control, our univariate Cox model indicated that increasing total dose was a significant predictor of higher in-field progression rates (HR = 2.97; *p* = 0.003). All 10 recurrences occurred in patients receiving doses between 55.8 and 56 Gy in 1.8–2.0 Gy per fraction. This finding presents a fascinating paradox, and while it may initially seem counterintuitive, it potentially reflects the unique radiobiology of benign tumors like VS. The primary goal of SRT is not to eradicate every cell but rather to induce terminal growth arrest through mechanisms like vascular endothelial and DNA damage, leading to reproductive cell death [25]. Excessive dose beyond what is required to trigger this arrest may not provide additional benefit and could theoretically incite a proinflammatory or profibrotic tumor microenvironment that, in rare instances, might paradoxically support regrowth or treatment resistance [26, 29]. This observation challenges historical data that suggested improved control with doses at the higher end of the spectrum [10] and aligns with a growing body of evidence indicating that moderate doses are sufficient for excellent control [30, 31]. For instance, Meijer et al. [30] found no significant difference in tumor control between biologically different fractionation schedules of 20 × 2.5 Gy (50 Gy) and 25 × 2 Gy (50 Gy), suggesting a plateau in the dose–response curve. Our data imply that for NFSRT, this plateau may be reached at or even below 55.8 Gy, and exceeding it could be detrimental. This warrants serious consideration for protocol adjustment, potentially adopting a lower dose range of 50–54 Gy for standard fractionation, which has been shown to be highly effective while potentially improving the therapeutic ratio [30, 32]. Adding to this, no significant differences were found regarding tumor volumes in that group, although our standard was to treat larger tumors with lower doses to allow better OAR sparing.

Hearing preservation remains one of the most critical quality of life metrics in VS management. Our cohort had a high baseline rate of useful hearing impairment (86.9%), which remained stable at final follow-up (88.0%). This indicates that while SRT is highly effective at halting tumor progression and preventing further neurological decline, it rarely reverses pre-existing auditory deficits. This underscores the critical importance of early intervention while hearing is still serviceable, a conclusion strongly supported by prospective studies [9]. The high baseline impairment rate in our study precludes definitive comparison of hearing preservation rates between SRS and NFSRT, as there was little functional hearing left to preserve in many patients. This is a common challenge in retrospective series. The ongoing debate regarding a potential advantage of fractionated regimens for hearing preservation, based on the radiobiological principle of a more favorable alpha/beta ratio for neural tissue, therefore remains unresolved by our data [11, 33]. Prospective trials specifically enrolling patients with good baseline hearing are needed to definitively answer this question.

The analysis of symptom evolution before and after SRT revealed a generally stable or improving picture for most neurological symptoms. The rates of facial paralysis (7.4% before to 6.9% after SRT) and trigeminal neuralgia (1.1% before to 2.9% after SRT) were low and stable, reinforcing the superior facial nerve preservation profile of SRT compared to historical surgical series showing rates of up to 38% of facial nerve affection [34, 35]. Regarding trigeminal function, SRS in our series seems equal to surgical reports [36, 37]. This excellent safety profile is a direct result of steep dose gradients and rigorous adherence to modern dose constraints for the cranial nerves [38]. Nonetheless, the observed associations of higher age with lower rates of vertigo or tinnitus should be interpreted cautiously. Given the low event rates and retrospective design, such findings may reflect chance variation, reporting bias, or incomplete documentation rather than true biological effects. These signals are best considered hypothesis generating and warrant confirmation in prospective studies with standardized symptom assessment.

However, it is notable that the headache prevalence rose from 14.3% at baseline to 22.3% at final follow-up (*p* = 0.02). Post-SRT headache is a well-documented, though not fully understood, phenomenon. Carlson et al. [6] reported a 50% incidence of headaches up to 8 years after VS SRT, noting that they can be a delayed effect. This underscores the necessity of thorough patient counseling prior to treatment, setting realistic expectations not only for acute effects but also for potential long-term quality of life changes. The management of these patients may benefit from a multidisciplinary approach involving neurologists or pain specialists.

The transient nature of acute toxicities like fatigue and alopecia—showing a higher association with NFSRT—which were significant at the first (T1) but had resolved by the final follow-up (T2), is an important counseling point. Patients opting for a fractionated course should be reassured that these side effects, while common during treatment, are typically self-limiting.

Our analysis revealed that patients who had undergone prior partial resection were more prone to reporting vertigo at the final follow-up (OR = 0.3; *p* = 0.005). This is an insightful observation that speaks to the complex interplay between treatment modalities and functional outcomes. Surgery in the cerebellopontine angle can cause vestibular disruption by damaging the vestibular nerve or its vascular supply [21]. This, combined with the subsequent effects of radiotherapy on any remaining vestibular function, may impair the brain’s ability to fully compensate, leading to persistent dizziness and imbalance. This finding reinforces the argument for primary SRT in eligible patients, as it may offer a better chance of preserving vestibular function and facilitating neural compensation compared to a combined-modality approach that introduces the traumas of both surgery and radiation [22]. It also highlights the paramount importance of dedicated vestibular rehabilitation programs for all VS patients, particularly those undergoing multimodality treatment, to promote central compensation and improve functional outcomes [22].

The very low incidence of radionecrosis (0.6%) in our series is testament to the advancements in treatment planning and delivery. The use of high-resolution MRI fusion (including CISS/FIESTA sequences for superior nerve delineation), precise immobilization with thermoplastic masks, and daily image-guided verification ensure highly conformal dose distributions that spare critical organs at risk. This result is consistent with modern literature, where the risk of symptomatic radionecrosis is consistently reported at below 2% when established dose–volume constraints for the brainstem are meticulously adhered to [23, 38]. Interestingly though, the differentiation between progression, pseudoprogression, and radionecrosis remains challenging and demands interdisciplinary efforts [16–18]. In sum, the excellent safety profile makes SRT a very attractive option for patients.

The interpretations of this study must be contextualized within its limitations. The retrospective, single-institution design introduces inherent potential for selection bias and unmeasured confounding. While we statistically adjusted for factors like PTV size, other unaccounted-for variables may have influenced outcomes. The evolution of institutional techniques and dose prescriptions over the 25-year inclusion period is another confounding factor, reflecting the natural progression in the field but making direct comparisons over time less straightforward. The lack of standardized, prospectively collected quality of life data (e.g., using PANQOL or SF-36 questionnaires) is a significant limitation, as it restricts our analysis to physician-reported symptoms rather than patient-reported outcomes, which are increasingly recognized as paramount [24]. Furthermore, toxicity data were derived from long-term follow-up assessments, which may be subject to reporting and recall bias. Moreover, toxicity grading was not consistently harmonized (dichotomous documentation, VAS, or CTCAE), potentially limiting comparability and the statistical robustness of symptom analyses. Missing data at the first follow-up (T1) for some symptoms that were not routinely assessed at that time further complicates a complete understanding of the acute phase and insights into when symptoms form following SRT. Likewise, it would have been desirable to have had the PTV for all patients, in order to confidently assess the influence of the PTV on clinical outcomes. Lastly, we chose to analyze late toxicity as a fluid endpoint counted at the time of last FU. Therefore, the reported toxicity was assessed between 6 and 282 months after RT. This could introduce significant bias, as early-late and delayed-late toxicity may differ.

Despite these limitations, the study has considerable strengths, including a relatively large, well-characterized patient cohort; a robust median follow-up duration; and a detailed analysis of a wide array of symptoms and toxicities over time. The homogenous treatment within two clear modality groups (SRS and NFSRT) also adds clarity to the comparisons.

## Conclusion

Our study strongly reaffirms that both SRS and NFSRT are highly effective and safe primary or adjuvant treatments for vestibular schwannoma, providing outstanding long-term tumor control with a favorable toxicity profile. The paradoxical finding of a potentially inverse dose–response relationship for fractionated regimens challenges conventional wisdom and suggests a compelling opportunity for protocol de-escalation to further enhance patient safety without compromising efficacy. The persistence of certain symptoms like headache and vertigo, particularly after multimodality treatment, underscores the need for comprehensive patient counseling, proactive management, and the integration of functional rehabilitation. Ultimately, the choice of modality should be tailored to individual patient and tumor characteristics through a shared decision-making process, informed by the robust evidence base that this study contributes to.

## References

[1] Louis DN. The 2021 WHO Classification of Tumors of the Central Nervous System: a summary - PubMed [Internet]. [zitiert 20. Februar 2026]. Verfügbar unter: https://pubmed.ncbi.nlm.nih.gov/34185076/

[2] Howitz MF, Johansen C, Tos M, Charabi S, Olsen JH (2000) Incidence of vestibular schwannoma in Denmark, 1977–1995. Am J Otol 21(5):690–694 10993460

[3] Marinelli JP, Beeler CJ, Carlson ML, Caye-Thomasen P, Spear SA, Erbele ID (2022) Global Incidence of Sporadic Vestibular Schwannoma: A Systematic Review. Otolaryngol Neck Surg 167(2):209–214. 10.1177/01945998211042006 10.1177/01945998211042006 34464224

[4] Stangerup SE, Caye-Thomasen P (2012) Epidemiology and natural history of vestibular schwannomas. Otolaryngol Clin North Am 45(2):257–268. 10.1016/j.otc.2011.12.008 22483814 10.1016/j.otc.2011.12.008

[5] M K, K K, S H, T D, SE S, P C‑T (2017) Ten-Year Follow-up on Tumor Growth and Hearing in Patients Observed With an Intracanalicular Vestibular Schwannoma. Neurosurgery 80(1):49–56. 10.1227/NEU.0000000000001414 27571523 10.1227/NEU.0000000000001414

[6] Carlson ML, Link MJ (2021) Vestibular Schwannomas. N Engl J Med 384(14):1335–1348. 10.1056/NEJMra2020394 33826821 10.1056/NEJMra2020394

[7] Goldbrunner R, Weller M, Regis J, Lund-Johansen M, Stavrinou P, Reuss D et al (2020) EANO guideline on the diagnosis and treatment of vestibular schwannoma. Neuro-Oncol 22(1):31–45. 10.1093/neuonc/noz153 31504802 10.1093/neuonc/noz153 PMC6954440

[8] Milligan BD, Pollock BE, Foote RL, Link MJ (2012) Long-term tumor control and cranial nerve outcomes following γ knife surgery for larger-volume vestibular schwannomas. J Neurosurg 116(3):598–604. 10.3171/2011.11.JNS11811 22175724 10.3171/2011.11.JNS11811

[9] Murphy ES, Suh JH (2011) Radiotherapy for vestibular schwannomas: a critical review. Int J Radiat Oncol Biol Phys 79(4):985–997. 10.1016/j.ijrobp.2010.10.010 21353158 10.1016/j.ijrobp.2010.10.010

[10] Combs SE, Volk S, Schulz-Ertner D, Huber PE, Thilmann C, Debus J (2005) Management of acoustic neuromas with fractionated stereotactic radiotherapy (FSRT): long-term results in 106 patients treated in a single institution. Int J Radiat Oncol Biol Phys 63(1):75–81. 10.1016/j.ijrobp.2005.01.055 16111574 10.1016/j.ijrobp.2005.01.055

[11] Flickinger JC, Kondziolka D, Niranjan A, Lunsford LD (2001) Results of acoustic neuroma radiosurgery: an analysis of 5 years’ experience using current methods. J Neurosurg 94(1):1–6. 10.3171/jns.2001.94.1.0001 11147876 10.3171/jns.2001.94.1.0001

[12] Linskey ME (2008) Hearing preservation in vestibular schwannoma stereotactic radiosurgery: what really matters? J Neurosurg 109(Suppl):129–136. 10.3171/JNS/2008/109/12/S20 19123899 10.3171/JNS/2008/109/12/S20

[13] Andrews DW, Suarez O, Goldman HW, Downes MB, Bednarz G, Corn BW et al (2001) Stereotactic radiosurgery and fractionated stereotactic radiotherapy for the treatment of acoustic schwannomas: comparative observations of 125 patients treated at one institution. Int J Radiat Oncol Biol Phys 50(5):1265–1278. 10.1016/s0360-3016(01)01559-0 11483338 10.1016/s0360-3016(01)01559-0

[14] Eisenhauer EA, Therasse P, Bogaerts J, Schwartz LH, Sargent D, Ford R et al (2009) New response evaluation criteria in solid tumours: Revised RECIST guideline (version 1.1). Eur J Cancer 45(2):228–247. 10.1016/j.ejca.2008.10.026 19097774 10.1016/j.ejca.2008.10.026

[15] Fega KR, Fletcher GP, Waddle MR, Peterson JL, Ashman JB, Barrs DM et al (2019) Analysis of MRI Volumetric Changes After Hypofractionated Stereotactic Radiation Therapy for Benign Intracranial Neoplasms. Adv Radiat Oncol 4(1):43–49. 10.1016/j.adro.2018.08.013 30706009 10.1016/j.adro.2018.08.013 PMC6349623

[16] Fouard O, Daisne JF, Wanet M, Regnier M, Gustin T (2022) Long-term volumetric analysis of vestibular schwannomas following stereotactic radiotherapy: Practical implications for follow-up. Clin Transl Radiat Oncol 33:1–6. 10.1016/j.ctro.2021.12.003 34977365 10.1016/j.ctro.2021.12.003 PMC8688865

[17] Rueß D, Schütz B, Celik E, Baues C, Jünger ST, Neuschmelting V et al (2023) Pseudoprogression of Vestibular Schwannoma after Stereotactic Radiosurgery with Cyberknife®: Proposal for New Response Criteria. Cancers 15(5):1496. 10.3390/cancers15051496 36900290 10.3390/cancers15051496 PMC10000564

[18] Walker AJ, Ruzevick J, Malayeri AA, Rigamonti D, Lim M, Redmond KJ et al (2014) Postradiation imaging changes in the CNS: how can we differentiate between treatment effect and disease progression? Future Oncol Lond Engl 10(7):1277–1297. 10.2217/fon.13.271 10.2217/fon.13.271 PMC4325371 24947265

[19] (2024) Downloading IBM SPSS Statistics 29 [CT738,CT763,CT761,CT762] [Internet]. https://www.ibm.com/support/pages/downloading-ibm-spss-statistics-29. Accessed 19.9.2024

[20] Prism - GraphPad [Internet]. [zitiert 23. Juli 2025]. Verfügbar unter: https://www.graphpad.com/features

[21] Iwai Y, Yamanaka K, Ishiguro T (2003) Surgery combined with radiosurgery of large acoustic neuromas. Surg Neurol 59(4):283–289. 10.1016/s0090-3019(03)00025-9 12748011 10.1016/s0090-3019(03)00025-9

[22] Yap J, Palmer G, Graving K, Stone S, Gane EM (2024) Vestibular Rehabilitation: Improving Symptomatic and Functional Outcomes of Persons With Vestibular Schwannoma: A Systematic Review. Phys Ther 104(10):pzae085. 10.1093/ptj/pzae085 38982735 10.1093/ptj/pzae085 PMC11450271

[23] Bubeníková A, Vlasák A, Fík Z, Sedlák V, Tesařová M, Bradáč O (2023) Application of diffusion tensor imaging of the facial nerve in preoperative planning for large vestibular schwannoma: a systematic review. Neurosurg Rev 46(1):298–298. 10.1007/s10143-023-02214-x 37950058 10.1007/s10143-023-02214-x

[24] Shaffer BT, Cohen MS, Bigelow DC, Ruckenstein MJ (2010) Validation of a disease-specific quality-of-life instrument for acoustic neuroma: the Penn Acoustic Neuroma Quality-of-Life Scale. The Laryngoscope 120(8):1646–1654. 10.1002/lary.20988 20641085 10.1002/lary.20988

[25] Kondziolka D, Shin SM, Brunswick A, Kim I, Silverman JS (2015) The biology of radiosurgery and its clinical applications for brain tumors. Neuro-Oncol 17(1):29–44. 10.1093/neuonc/nou284 25267803 10.1093/neuonc/nou284 PMC4483054

[26] Farhood B, Khodamoradi E, Hoseini-Ghahfarokhi M, Motevaseli E, Mirtavoos-Mahyari H, Eleojo Musa A et al (2020) TGF‑β in radiotherapy: Mechanisms of tumor resistance and normal tissues injury. Pharmacol Res 155:104745. 10.1016/j.phrs.2020.104745 32145401 10.1016/j.phrs.2020.104745

[27] Marchetti M, Pinzi V, Gemma M, Cuccarini V, Pascuzzo R, Cane I, Romeo A, Morlino S, De Martin E, Fariselli L. Hypofractionated Versus Single-Session Radiosurgery to Preserve Hearing in Patients Affected by Sporadic Vestibular Schwannoma: The ACOUNEU Randomized Clinical Trial. Int J Radiat Oncol Biol Phys. 2025 Sep 1;123(1):107-117. 10.1016/j.ijrobp.2025.03.081. Epub 2025 Apr 11. PMID: 40222395. 10.1016/j.ijrobp.2025.03.081 40222395

[28] Gawish A, Walke M, Röllich B, Ochel HJ, Brunner TB. Vestibular Schwannoma Hypofractionated Stereotactic Radiation Therapy in Five Fractions. Clin Oncol (R Coll Radiol). 2023 Jan;35(1):e40-e47. 10.1016/j.clon.2022.10.014. Epub 2022 Nov 2. PMID: 36335041. 10.1016/j.clon.2022.10.014 36335041

[29] Verginadis II, Citrin DE, Ky B, Feigenberg SJ, Georgakilas AG, Hill-Kayser CE et al (2025) Radiotherapy toxicities: mechanisms, management, and future directions. Lancet Lond Engl 405(10475):338–352. 10.1016/S0140-6736(24)02319-5 10.1016/S0140-6736(24)02319-5 PMC12758832 39827884

[30] Meijer OWM, Vandertop WP, Baayen JC, Slotman BJ (2003) Single-fraction vs. fractionated linac-based stereotactic radiosurgery for vestibular schwannoma: a single-institution study. Int J Radiat Oncol Biol Phys 56(5):1390–1396. 10.1016/s0360-3016(03)00444-9 12873685 10.1016/s0360-3016(03)00444-9

[31] Fuss M, Debus J, Lohr F, Huber P, Rhein B, Engenhart-Cabillic R et al (2000) Conventionally fractionated stereotactic radiotherapy (FSRT) for acoustic neuromas. Int J Radiat Oncol Biol Phys 48(5):1381–1387. 10.1016/s0360-3016(00)01361-4 11121637 10.1016/s0360-3016(00)01361-4

[32] Sawamura Y, Shirato H, Sakamoto T, Aoyama H, Suzuki K, Onimaru R et al (2003) Management of vestibular schwannoma by fractionated stereotactic radiotherapy and associated cerebrospinal fluid malabsorption. J Neurosurg 99(4):685–692. 10.3171/jns.2003.99.4.0685 14567604 10.3171/jns.2003.99.4.0685

[33] van Leeuwen CM, Oei AL, Crezee J, Bel A, Franken NAP, Stalpers LJA et al (2018) The alfa and beta of tumours: a review of parameters of the linear-quadratic model, derived from clinical radiotherapy studies. Radiat Oncol 13(1):96. 10.1186/s13014-018-1040-z 29769103 10.1186/s13014-018-1040-z PMC5956964

[34] Régis J, Pellet W, Delsanti C, Dufour H, Roche PH, Thomassin JM et al (2002) Functional outcome after gamma knife surgery or microsurgery for vestibular schwannomas. J Neurosurg 97(5):1091–1100. 10.3171/jns.2002.97.5.1091 12450031 10.3171/jns.2002.97.5.1091

[35] Rinaldi V, Casale M, Bressi F, Potena M, Vesperini E, De Franco A et al (2012) Facial Nerve Outcome after Vestibular Schwannoma Surgery: Our Experience. J Neurol Surg Part B Skull Base 73(1):21–27. 10.1055/s-0032-1304559 10.1055/s-0032-1304559 PMC3424019 23372991

[36] Nonaka Y, Fukushima T, Watanabe K, Friedman AH, Sampson JH, Mcelveen JTJ et al (2013) Contemporary Surgical Management of Vestibular Schwannomas: Analysis of Complications and Lessons Learned Over the Past Decade. Oper Neurosurg 72:ons103. 10.1227/NEU.0b013e3182752b05 10.1227/NEU.0b013e3182752b05 23037828

[37] Betka J, Zvěřina E, Balogová Z, Profant O, Skřivan J, Kraus J et al (2014) Complications of Microsurgery of Vestibular Schwannoma. BioMed Res Int 2014:315952. 10.1155/2014/315952 24987677 10.1155/2014/315952 PMC4058457

[38] Kano H, Kondziolka D, Khan A, Flickinger JC, Lunsford LD (2009) Predictors of hearing preservation after stereotactic radiosurgery for acoustic neuroma. J Neurosurg 111(4):863–873. 10.3171/2008.12.JNS08611 19284227 10.3171/2008.12.JNS08611

